# Assessing the Influence of Sensor-Induced Noise on Machine-Learning-Based Changeover Detection in CNC Machines

**DOI:** 10.3390/s24020330

**Published:** 2024-01-05

**Authors:** Vinai George Biju, Anna-Maria Schmitt, Bastian Engelmann

**Affiliations:** Institute of Digital Engineering, Technical University of Applied Sciences Wuerzburg-Schweinfurt, 97421 Schweinfurt, Germany; vinai.georgebiju@study.thws.de (V.G.B.);

**Keywords:** sensor noise, LightGBM, machine learning, NC sensor data, CNC

## Abstract

The noise in sensor data has a substantial impact on the reliability and accuracy of (ML) algorithms. A comprehensive framework is proposed to analyze the effects of diverse noise inputs in sensor data on the accuracy of ML models. Through extensive experimentation and evaluation, this research examines the resilience of a LightGBM ML model to ten different noise models, namely, Flicker, Impulse, Gaussian, Brown, Periodic, and others. A thorough analytical approach with various statistical metrics in a Monte Carlo simulation setting was followed. It was found that the Gaussian and Colored noise were detrimental when compared to Flicker and Brown, which are identified as safe noise categories. It was interesting to find a safe threshold limit of noise intensity for the case of Gaussian noise, which was missing in other noise types. This research work employed the use case of changeover detection in (CNC) manufacturing machines and the corresponding data from the publicly funded research project (OBerA).

## 1. Introduction

(ML) techniques are increasingly finding their way into today’s production systems. Training these models requires sensor data recorded in a production environment. The sensors used are exposed to a wide variety of interferences, which become noticeable as noise in the measurement data.

Sensor-induced noise can influence ML models, and it is critical to assess how sensor data can then be utilized to optimize error when predicting a changeover of the machining process [1]. It was observed that data augmented with Gaussian noise improved a prediction model using ML [2]. Gaussian noise was also used in the generator part of (GAN) models to generate new data samples [3].

ML practitioners should be able to diagnose and quantify the effects of sensor-induced noise and improvise to accommodate the noise characterization [4]. Inaccurate sensor data can cause costly errors, machining downtimes, and misplanned machining operations [5]. An ML-based chatter detection system that utilized machining process audio recordings to augment periodic noise was designed [6]. A convolutional auto-encoder model was built to fuse multi-sensor data and suppress the effects of noises in CNC machine tool data, resulting in superior unsupervised anomaly detection performance [7].

In this study, an ML approach and corresponding real-world data, which were acquired in the research project (OBerA) to realize the automatic detection of changeover events in manufacturing machines, were used. The project was formed to assist metalworking companies from the region of Franconia in northern Bavaria in the digitization of their system landscape.

The overheads that arise out of erroneous sensor data can then be placed under control by analyzing the impacts of different types of noises and implementing operational noise reduction techniques. The initial hypothesis is that the reliability and accuracy of an ML model in detecting changeovers by utilizing an external sensor and (NC) data are significantly affected by the different types of induced noise in the data.

The following research questions shall be answered in this study:

1. How are the accuracy and reliability in changeover detection affected by the sensor-induced noise in an ML system framework?
2. To what degree can ML models effectively detect changeovers in industrial processes when there is sensor-induced noise?
3. Can sensor-induced noise be successfully handled and reduced to ensure accurate and trustworthy changeover detection?
4. Is it possible to define a safe threshold value or intensity for each noise type in the data that affects the accuracy of the ML soft sensor?

To answer these questions, the LightGBM ML algorithm and the effects of Gaussian, Brown, Uniform, Salt-and-Pepper, Impulse, Flicker, Multiplicative, Periodic, 1/f, and Colored noise were evaluated in Monte Carlo simulation settings.

This article is structured as follows: Section 2 introduces the ML model as a soft sensor and explains the measurement uncertainty. An overview of the research can be found in Section 3. The experimental setup for detecting changeovers in CNC machines is mentioned in Section 4.1. It is followed by a description of the key noise types used in the study and their characteristics in Section 4.3. The LightGBM soft sensor framework used in a Monte Carlo setting is explained in Section 4.4. Section 5 describes the results derived from the LightGBM model and presents a further analysis in terms of outlier dispersion, kernel density estimations, and swarm analysis. It also describes the variations in accuracy and (FPR) with respect to the changing intensity of different noise types. The practical and strategic findings from the study regarding the classification of the noise types and understanding of the noise intensity threshold are mentioned in Section 6.

## 2. Machine Learning Model as Soft Sensor

To understand the influence of noise on an ML model, it is valuable to revisit the concept of soft sensors: A soft sensor is a virtual sensor that estimates or predicts physical variables or process parameters based on available measurements [8]. In the context of (CNC) machines, an ML model can be trained on historical data collected from sensors within the machine to learn patterns and relationships between input variables (such as cutting parameters, tool wear, and material properties) and output variables (such as surface quality, tool life, and machining time). ML models then act as soft sensors that use data from sensors attached to the (NC) of a machine or by means of external sensors.

### 2.1. Measurement Uncertainty

It is critical to assess and evaluate the measurement uncertainty in sensor-based data in industrial manufacturing. Measurement errors are mainly categorized as systematic and random measurement deviations. Random measurement deviations constantly add to the measurement uncertainty and are difficult to correct or detect. The noise in data from sensors in a machining environment contributes to random measurement deviations. The continual non-ideal behavior of sensor signal processing is the cause of systematic measurement discrepancies. Calibration measurements, which are also known as comparison measurements, are a useful tool for identifying them. The calibration of sensors and correction measurements in software are remedial measures against measurement uncertainty, leaving a minor residual variance in the calibration process [9].

The sensors used on the shop floor are expected to produce precise measurements to guarantee accurate control and monitoring of the CNC machine production life cycle. The quality standards in sensor measurements need to be sustained to accomplish the desired manufacturing outcomes [10].

Extreme environmental conditions during manufacturing, such as vibration, temperature, and humidity, need to be tolerated by sensors for the data that are generated to be reliable without degrading the sensors’ function. The sensors ought to be highly sensitive to even minor changes in the parameters being monitored. Upon timely activation, remedial actions can result in the early detection of abnormalities or deviations from anticipated values in the data [11].

### 2.2. Uncertainty Budget

An uncertainty budget’s objective is to accurately assess measurement uncertainty using a well-structured systematic process with the (GUM) method. The data derived from sensors can then have a formal record of the uncertainty analysis procedure and be formally authenticated by experts in the field. The investigation and reporting of all important sources of uncertainty that significantly affect a sensor’s total measurement uncertainty constitute the uncertainty budget of a sensor used in industrial manufacturing [12].

Type A uncertainty is evaluated via statistical analysis using data from comparable sources or repeated measurements. This includes analyzing the measurement variability along with assessing uncertainties with the use of statistical techniques such as the standard deviation or confidence intervals. Type B uncertainty is evaluated in several ways other than the statistical analysis of measurable data. This entails estimating uncertainties from sources that have been established, such as calibration certifications and specifications offered by a sensor’s manufacturer or professional expertise [13].

### 2.3. Ishikawa Analysis of a Machine Learning Model as a Soft Sensor

**Figure 1:**
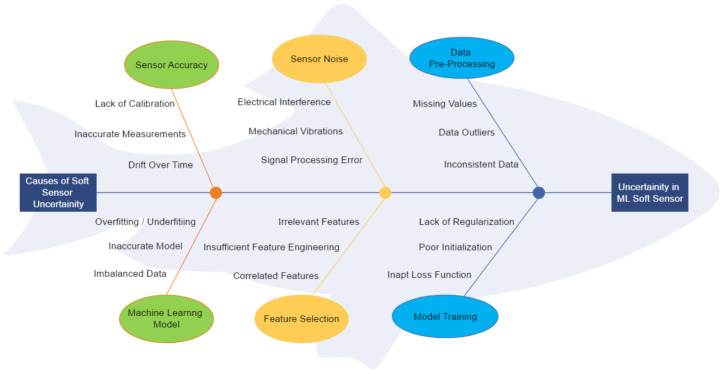
Cause and effect: uncertainty of an ML model as a soft sensor.

A cause-and-effect diagram, as shown in Figure 1, is a general tool for linking effects to causes. It is often applied to uncertainty budgets for measurement devices to identify the most important contributors to the uncertainty in the data captured by a sensor [14]. Generally, the diagram is filled out by experts from the domain or derived through literature research. It helps to identify and visualize the root causes of issues or challenges in evaluating uncertainty by using an ML model as a soft sensor.

The main causes include sensor accuracy, sensor noise, data preprocessing, ML models, feature selection, and model training, which have a direct impact on the ML task. The three most important underlying causes in each category are listed in the Ishikawa analysis in Figure 1. The performance of the ML model is affected by sensor noise, according to the Ishikawa analysis. A couple of the leading causes that impair the sensor data with noise are the environment of the CNC machine and electrical interference. The practitioner investigating the uncertainty in a prediction using an ML model should be able to identify the noise types and their impacts on the accuracy and precision of the model. The resistance of the model to changes and uncertainties in the actual world can be reinforced by adding different types of noise patterns to sensor data during model training. The ML soft sensor’s uncertainty in predictions can substantially increase due to complex noise forms and data sampling rates [15,16].

Varying noise levels or ambiguous sensor readings may have a substantial impact on an ML model’s performance. The model adapts and becomes more generalized with exposure to a broader range of noise levels and patterns. A scenario that does not consider the noise parameters could result in overfitting, where the model becomes overly specialized for the training data and, thereby, suffers in generalizing to new data [17].

The practitioner needs to be aware that noise during training might have advantages. Rather than relying on individual noise-free data from the machining setting, the noise types can cause the model to capture more generic patterns and relationships. The induction of noise in the data can be used as a method of data augmentation to broaden the range and spectrum of training samples. The model can gain insight from a wider variety of cases and enhance its overall prediction capabilities, thereby imitating specific challenges or abnormalities that appear in sensor data collected in actual machining settings [18].

## 3. Overview of the Research Topic

There are two main possibilities for designing a solution for changeover process prediction. In Possibility 1, an ML model is trained without noise types induced in the data. Since ML models are often referred to as “black boxes”, it might be difficult to comprehend how the model arrives at its particular prediction for the changeover. The uncertainty in forecasts holds, especially when dealing with data from sensors and assuming the non-existence of noise in the data. The focus is on studying and experimenting with Possibility 2, where an ML model is trained with noise types induced in the data.

**Figure 2:**
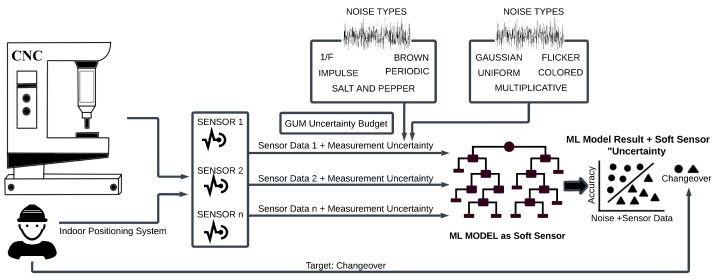
Architecture design: changeover detection with sensor noise.

According to Figure 2 and Nettleton et al. [4], noise was applied to the sensor data for the training and test datasets (input variable). As the output variable was a binary status variable, no noise was applied there.

This approach allows the ML model to act as a soft sensor and to interpret the behavior and type of the input sensor data, making it suitable for handling complex relationships with the changeover class variable (see Section 4.2). The uncertainty in the processed data from sensors is to be verified to understand its variability. Soft-sensor-based ML techniques must be designed to accommodate these uncertainties and to improve the accuracy in prediction tasks. To understand the possible source or nature of noise embedded in the data, data from sensors need to be cross-checked. Noise patterns in the data can introduce random spikes or be found to be periodic.

The data were separately induced with the following types of noise: Flicker, Gaussian, Uniform, Periodic, 1/f, Colored, Multiplicative, Salt and Pepper, Brown, and Impulse. Each noise type originates from specific environmental conditions and is also different in terms of the nature of noise visible in the data. The key architecture of the work is summarized in Figure 2. The measurement uncertainties of sensors due to the induction of noise in the data before training the ML model were simulated.

## 4. Materials and Methods

The architecture, as described in Figure 2, was implemented as follows: The data capture from the NC and external indoor positioning system was set up in conjunction with the HEIDENHAIN and Cybus Connectware machine interface. The uncertainty modeling with sensor-induced noise types was experimented with using a LightGBM ML soft sensor. The specifications of the CNC machine and the sensor setup are described in Section 4.1. Section 4.2 explains the changeover use case and its significance in the manufacturing environment. The description of the noise types used in the analysis can be found in Section 4.3. Section 4.4 describes the action of LightGBM as a soft sensor in a Monte Carlo simulation setting for the classification of changeovers.

### 4.1. Experimental Setup

**Figure 3:**
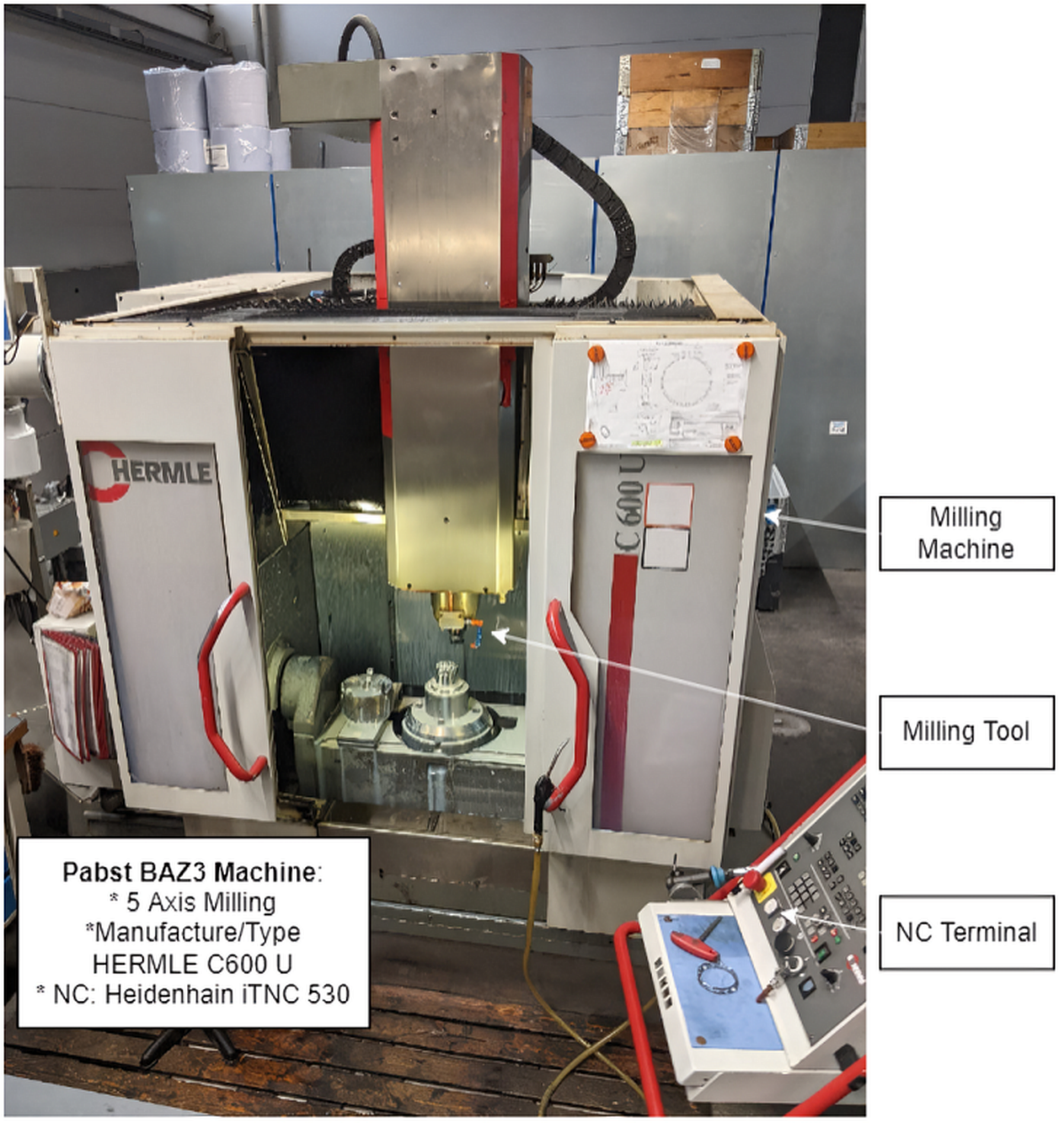
HERMLE C600 U milling machine with sensors.

All of the NC data were acquired from a milling machine, HERMLE C600 U, at the company Pabst Komponentenfertigung GmbH from the OBerA project consortium. The specific machine used in this study is shown in Figure 3. The CNC machine had 5-axis kinematics and an NC HEIDENHAIN iTNC 530, which was compatible with the HEIDENHAIN DNC interface. An Ethernet switch was utilized to link the NC and the NUC mini PC. An SQL database received the data after they had been processed by the Node-RED programming interface. The HEIDENHAIN DNC enabled communication between an agent on the PC and the NC. The MQTT protocol was used to transmit the data from the agent onto a Cybus Connectware interface. The Cybus interface exported these internal variables, such as door switches, through the HEIDENHAIN DNC interface, making these characteristics accessible.

Different feature variables from NC and indoor positioning sensors were acquired (see Table 1). Eight variables from the NC and two from an indoor positioning system as an external sensor (No. 9 + 10) were recorded. The feature variables used to induce noise in this study included OverrideFeed, FeedRate, SpindleSpeed, and Indoor GPSx/y. The noise intensity was the same throughout all five numerical variables mentioned above. The target variable, which was to be detected by the ML model, was the changeover status (status code: 0 for changeover, 1 for production) [19].

The indoor positioning system Localino is made by the company Heuel and Löher GmbH and Co. KG (Lennestadt, Germany) and uses ultra-wideband signals and a two-way ranging algorithm to determine the position of a worker on the shop floor on the *x*- and *y*-axes [9]. The data collected from sensors used in the analysis had 21,940 instances (rows) and ten feature variables, as described in Table 1. The ratio between training and test data was 80:20.

### 4.2. Use Case Description: Changeover Detection

When a machine is prepared and set up for the manufacture of a different product variety, a transition event occurs during the process of manufacturing, and this is referred to as a changeover [20]. For cost estimates, production sequencing, and employment schedules, highly accurate changeover timetables must be maintained.

On a factory floor, changeovers between manufacturing orders occur often, and they establish the production setting for a new manufacturing job category. Only prototypes are manufactured during the changeover process to ensure that the manufacturing process can be carried out successfully and that the process configuration is appropriate for the new product type that is scheduled. As a result, changeovers are seen as a production deficit [21].

Lean management techniques aim to reduce changeover times, which has been a critical task for enterprises. The changeover process takes place at the start of a production order and affects the total throughput time [19,22].

An ML model from previous research work was applied here to classify two phases of the changeover process of a CNC milling process based on information gathered from an external indoor positioning system, along with information from its (NC) system. The data were recorded live with the “2-phase concept”, where the states of “machine is in changeover” and “machine is in production” could be distinguished by the ML model.

### 4.3. Noise Types Induced in the Data

The sensors used in a manufacturing setup are susceptible to external influences and may generate undesirable disruptions in the captured data. The quality of the sensor data can be greatly impacted by these disturbances, and this was simulated in the actual data by using the induction of noise types. The study of noise induced in sensor data enabled us to analyze the form and effects of noise caused by various environmental conditions and glitches in sensors.

An Ishikawa analysis was used to determine the fundamental causes of diminishing accuracy in LightGBM as a soft sensor, which may have included elements that influenced measurement errors and uncertainty arising from noise. In accordance with GUM, the measurement uncertainty of the ML model as a soft sensor was quantified in order to guarantee the dependability and accuracy of measurement outcomes in the presence of noise parameters.

By understanding different noise forms, experts may use techniques that reduce their impacts and improve the accuracy and dependability of sensor data, resulting in more robust predictions from the ML model, e.g., filtering the noise from tool wear with a low-pass filter [23]. The induced noise can appear in numerous formats, each with its own features and impacts on the data quality. Noise can be caused by different sources, e.g., from mechanical vibrations and electrical and thermal fluctuations in a CNC machine during manufacturing [24]. Appendix A discusses the primary noise types used in this study, which may emerge from sensors within a CNC machine environment.

Table 2 compares the noise types that are described in Appendix A. The sources and possibilities for observation of each noise type are summarized in the second column. In the third column, information on how the specific noise type can be characterized is given. Common mitigation strategies for each noise type from the literature are listed in the last column. The parameters for each noise type and the corresponding values are mentioned in Appendix B.

### 4.4. Changeover Classification Using LightGBM

In the current research, it was determined that Random Forest algorithms are well suited for the classification task of the changeover detector [19]. In [57], it was shown that gradient-boosting algorithms such as LightGBM are also suited for ML tasks when Random Forest algorithms perform well. Gradient-boosting algorithms also offered advantages in the extensive simulations described in this article due to their fast speed in the training process and low memory utilization.

**Figure 4:**
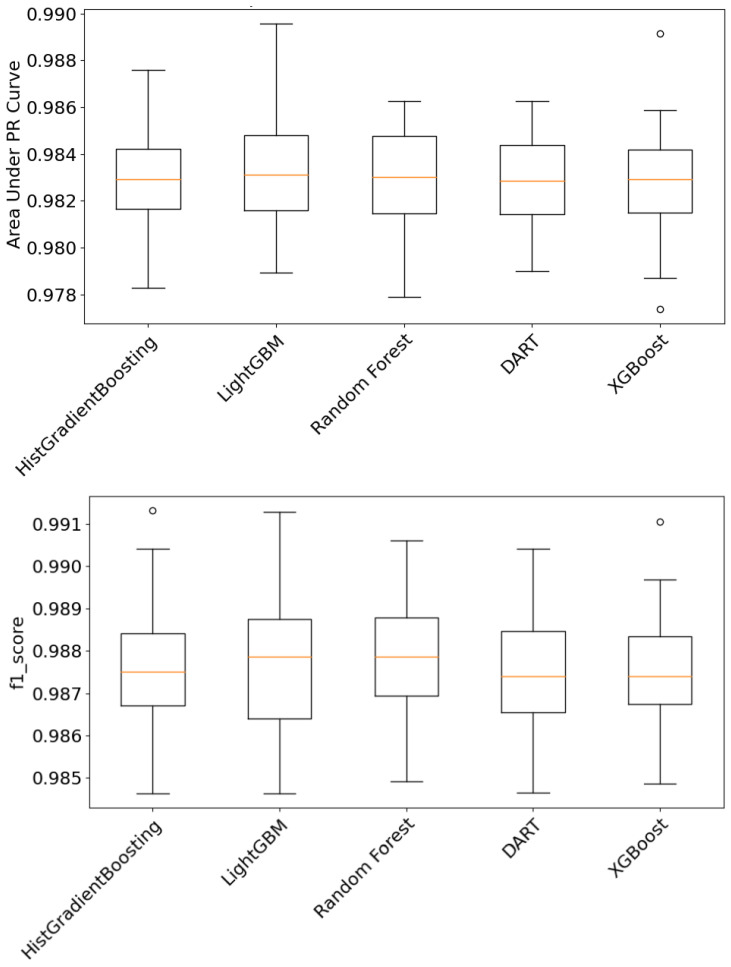
AUC-PR (**Top**) and F1 score (**Bottom**): comparison of changeover classification.

The boosting family of algorithms is less susceptible to outliers, can handle a variety of categorical and numerical variables without considerable preprocessing, and is scalable with efficient and parallelized implementations for diverse classification problems [58]. The HISTGradientBoosting [59], LightGBM, Random Forest [60], DART [61], and XGBoost [62] algorithms were compared to find the best ML soft sensor that was most appropriate for this case study. A box plot, which is shown in Figure 4, was used to reflect the probable outliers, central tendency, and dispersion for the HISTGradientBoosting, LightGBM, Random Forest, DART, and XGBoost algorithms in predicting changeovers in the presence of Gaussian noise. The (AUC-PR) metric indicated that LightGBM outperformed all other classification techniques, as shown in Figure 4 (Top). The median and upper quartile for the F1 score were better for LightGBM when compared to Random Forest and other algorithms in the boosting family, as shown in Figure 4 (Bottom).The evaluation had outlier values visible as a circle for XGBoost for AUC-PR curve metric and F1 score. The HistGradientBoosting also had a similar outlier value for the F1 Score.

To summarize, the LightGBM and Random Forest algorithms showed superior performance in the prediction modeling of changeovers in comparison with other algorithms in the boosting family. However, LightGBM was preferred over the Random Forest algorithm due to its speed and faster convergence in the training process. LightGBM was far quicker to model both in the Monte Carlo setting and in the experiment for determining the effect of accuracy while changing the intensity of noise. In conclusion, LightGBM worked best for acting as an ML soft sensor for practitioners in comparison with the other boosting algorithms when detecting changeovers.

The further application of neural networks for changeover detection will be the subject of future research.

**Figure 5:**
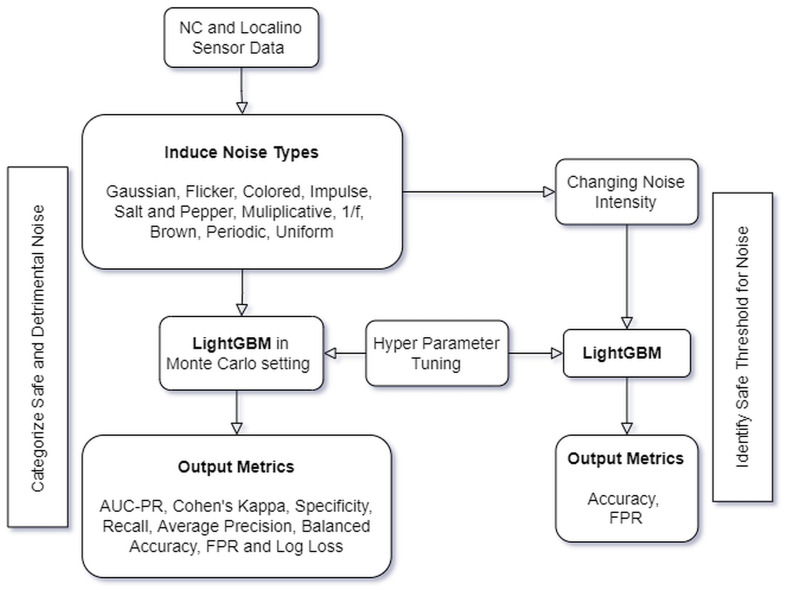
Noise-induced LightGBM framework.

LightGBM is known for its efficacy and superior performance due to its gradient-boosting architecture and traditional tree-based approaches to learning. It is crafted to handle large datasets and feature spaces with high dimensions while consuming the least memory and training time possible [63]. The ensemble of decision trees in LightGBM learns from the errors of the previous iteration in the training process. LightGBM extends the tree leaf-wise and prioritizes the nodes that give the most potential information gain by choosing the leaf node that reduces the loss at each split. This method often produces a more condensed tree, capturing more intricate data patterns. LightGBM uses histogram-based learning, which improves training efficiency and memory consumption. It creates histograms for discrete attributes in the dataset rather than sorting and binning the data points. The gradient-based histogram approach identifies optimal split points by computing the gradients of the loss function for every observation [64]. LightGBM uses a feature bundling strategy that groups relevant features to handle the dimensionality of data. Categorical features can be handled by LightGBM using a technique known as gradient-based one-side sampling [65]. The data from sensors for analyzing changeovers have categorical attributes, namely, ProgramStatus, ToolNumber, and PocketTable, as shown in Table 1. The flow of the work of understanding the effects of noise types on the performance of LightGBM is depicted in Figure 5.

#### 4.4.1. Hyperparameter Tuning in LightGBM

A grid or random search approach for hyperparameter tuning is generally defined as the use of a range of values to evaluate the optimal parameters of the model. While random search is more effective but may not come up with the optimal combination, grid search is comprehensive but computationally challenging [66]. Numerous validation sets using cross-validation for an exhaustive assessment of the model were utilized. The hyperparameters to be adjusted included the learning rate and the iterations of boosting cycles. An early stop parameter was used to reduce the search space of the model. The overfitting of the trees could be controlled by the max-depth of a tree and the min-child-samples in a leaf. The fine-tuning of the bagging fraction and feature fraction parameters in each boosting round could increase the model’s resilience. Additional hyperparameters, such as num-leaves, min-split-gain, and min-data-in-leaf, could be generally optimized depending on the specific scenario and dataset [67]. The residuals represent the inaccuracies in the predictions made by the model. The first derivative of the loss function with respect to the residuals was used to compute the gradient of the loss function. The derivative determined the rate at which the loss varied in response to the predictions, and the loss function governed the form of the gradient. The model generation involved evaluating the predictions in the direction that minimized loss by updating the gradient fraction [68].

#### 4.4.2. Monte Carlo Simulation for LightGBM

A Monte Carlo simulation framework for LightGBM that generated multiple prediction outcomes by picking samples from sensor-based data and altering the parameter settings that accounted for uncertainty was used. It could illustrate numerous realizations in the LightGBM setting that were best feasible in an uncertain scenario. It could also verify the robustness of the results from LightGBM and evaluate its performance with multiple random data samples. The Monte Carlo setting enabled the ML model to categorize the noise types into the categories of safe and detrimental after evaluating the statistical metrics resulting from changeover prediction for each noise type. In order to determine the best configuration of parameters for LightGBM, Monte Carlo simulations are a powerful means of exploring hyperparameter spaces by evaluating a wide variety of combinations and settings [69].

#### 4.4.3. Output Metrics for LightGBM

The output metrics used in the evaluation of LightGBM’s sensitivity to noise included the following: AUC-PR, Cohen’s Kappa, specificity, recall, average precision, balanced accuracy, FPR, and log loss. When dealing with classification tasks—especially with unbalanced datasets—AUC-PR offers crucial statistical insight into the efficacy of LightGBM while limiting false positives. In this study, the production class of sensor data exhibited an imbalance with nearly 1500 instances. Cohen’s Kappa provided a reliable statistic in which a Kappa score higher than the null value indicated that the model performed better than arbitrary guessing and offered a trustworthy indicator of accuracy. The specificity metric refers to the model’s capacity to recognize true negatives and is crucial in reducing the Type I error [70].

A lesser percentage of false positives is indicated by a higher value of specificity. The model’s ability to accurately detect true positives is measured with the recall or sensitivity and is significant in detecting changeovers. The precision–recall curve is summarized by the average precision, which offers a statistic for assessing the model’s capacity to balance precision and recall at various prediction boundaries. The balanced accuracy averages the sensitivity and specificity to regulate class imbalances in the data and offers a fair evaluation of the effectiveness of the model. The proportion of genuine negative classes that are mistakenly labeled as positive is measured with the FPR. The difference between the actual class values and the predicted class in the model is quantified by the log loss, which is additionally referred to as cross-entropy [71].

## 5. Results

**Figure 6:**
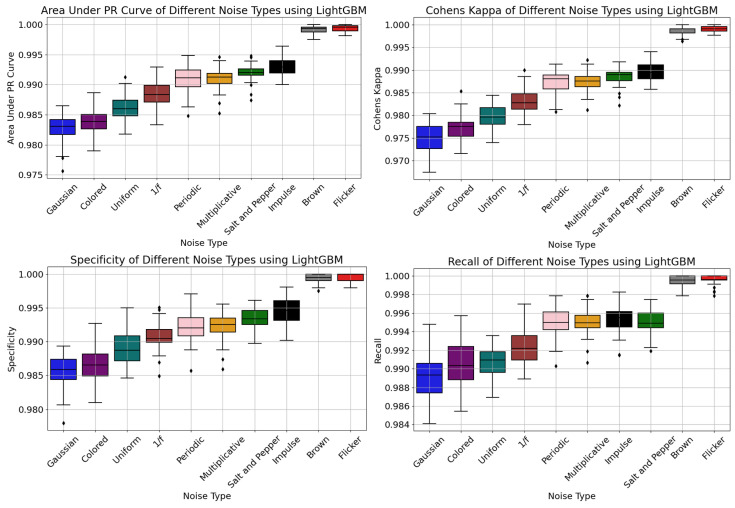
Outlier and dispersion analysis for multiple noise types.

The change in the level of accuracy of LightGBM based on the intensity of noise was verified using several output metrics—AUC-PR, Cohen’s Kappa, specificity, and recall—that were affected by various noise types—namely, Gaussian, Brown, Uniform, Salt-and-Pepper, Impulse, Flicker, Multiplicative, Periodic, 1/f, and Colored noise—as shown in Figure 6. It is evident from the results that the LightGBM model performed very well in the presence of Flicker and Brown noise, indicating that these two noise models had the least effect on the performance in predicting the changeover process.

The AUC-PR values in the presence of Flicker noise ranged from 0.998 to 1.0, which suggested that the soft-sensor-based LightGBM model performed extremely well in distinguishing the changeover outcomes despite the noise. The AUC-PR values in the presence of Gaussian noise using the LightGBM model varied between 0.976 and 0.987, resulting in a comparatively lower performance, causing minor ambiguity in the outcome variable. The Uniform, Gaussian, Periodic, Multiplicative, and Salt-and-Pepper noise produced outlier values in the AUC-PR analysis.

The uncertainty in the analysis that assessed the agreement between the LightGBM model’s predictions and the actual labels in changeovers was reflected through Cohen’s Kappa.

**Figure 7:**
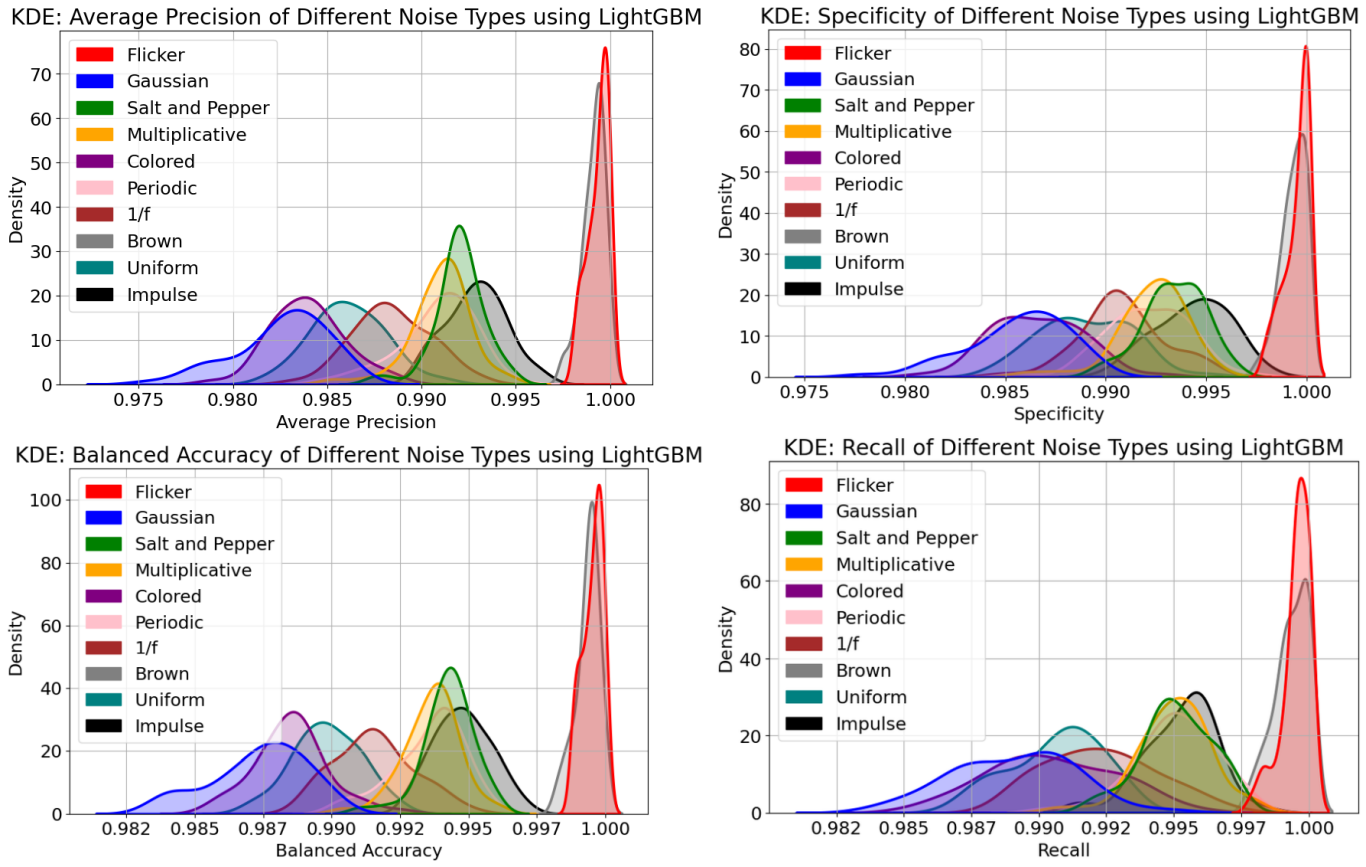
(KDE) of multiple noise types.

The model suffered the highest uncertainties in the presence of Gaussian and Colored noise when compared to other noise types, which was revealed by the lower values of Cohen’s Kappa. The results were similar when analyzing the true negative rate and true positive rate using specificity and recall to understand the effects of noise on the correct classification of negative and positive changeover classes. As demonstrated by Cohen’s Kappa, specificity, recall, and AUC-PR in Figure 6, the experiment demonstrated the beneficial impact of Flicker noise when training the LightGBM model. Outliers were identified for the Flicker noise while evaluating the recall and for the 1/f noise while evaluating the specificity and Cohen’s Kappa.

(KDE) plots were used to visualize the distribution of the average precision, balanced accuracy, specificity, and recall metrics, as shown in Figure 7. In particular, evaluating the kurtosis and skewness of the distributions across multiple noise scenarios can be useful for model adaptations.

Average precision was used to estimate the AUC-PR and provided insight into the ability of the LightGBM model to rank positive samples correctly. The Gaussian and Colored noise had the lowest average precision values and were observed to have a more skewed distribution than other noise types.

The Flicker and Brown noise had the best values for average precision with positive kurtosis.

The KDE metric for balanced accuracy considered both true positive and true negative rates and was appropriate for further analysis, since the changeover outcome class variable was slightly unbalanced. The KDE distribution for specificity and recall suggested that the LightGBM model’s effectiveness in appropriately identifying negative and positive changeover samples had a direct causal impact from the induced noise types. This highlighted the necessity of careful evaluation of the particular noise characteristics and the noise mitigation strategies in the changeover data. The overall KDE distribution suggested that the Gaussian noise had lower average precision, balanced accuracy, specificity, and recall values than those of the other noise types. Figure 7, similarly to Figure 6, shows that Gaussian and Colored noise were the worst noise types, while Flicker and Brown noise had the least impact on the model’s performance.

**Figure 8:**
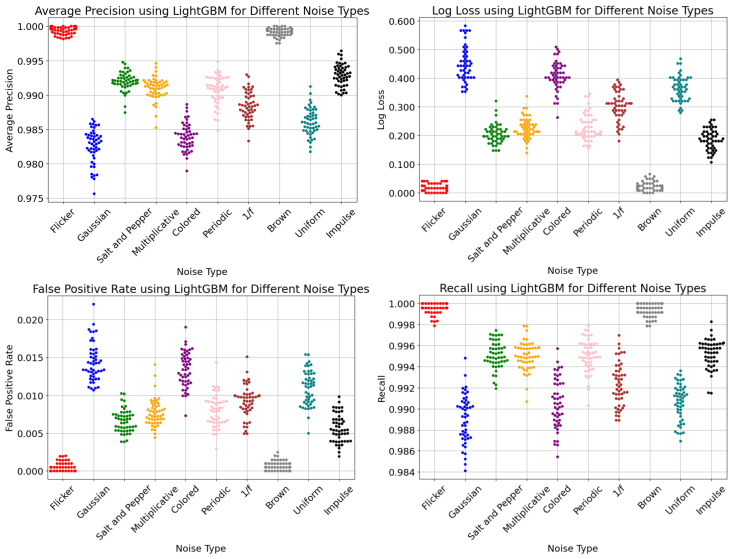
Swarm distributions of multiple noise types.

Swarm distribution plots, which are shown in Figure 8, offer a useful depiction of the LightGBM model’s performance, making it simple to compare the metric distributions across noise types. The average precision, FPR, log loss, and recall showed a compact swarm distribution pattern for Flicker and Brown noise. In contrast, Gaussian, Colored, and 1/f noise exhibited a more extended spread of swarm clusters, particularly in the recall values. Notably, Salt-and-Pepper, Multiplicative, and Periodic noise displayed relatively similar swarm clusters of metric values. The Flicker and Brown noise were found to have the lowest FPR in the range of 0 to 0.003. The Gaussian noise had the highest FPR, with values in the range of 0.011 to 0.024. It measured the rate at which the LightGBM model misclassified the negative changeover class as positive. This was crucial because the number of erroneous alerts in changeovers must be kept to a minimum to enhance productivity. The log loss score also showed a similar pattern to that of the FPR, as indicated in the swarm plot. A lower value for the log loss indicated a higher confidence level in the LightGBM model’s predictions.

**Figure 9:**
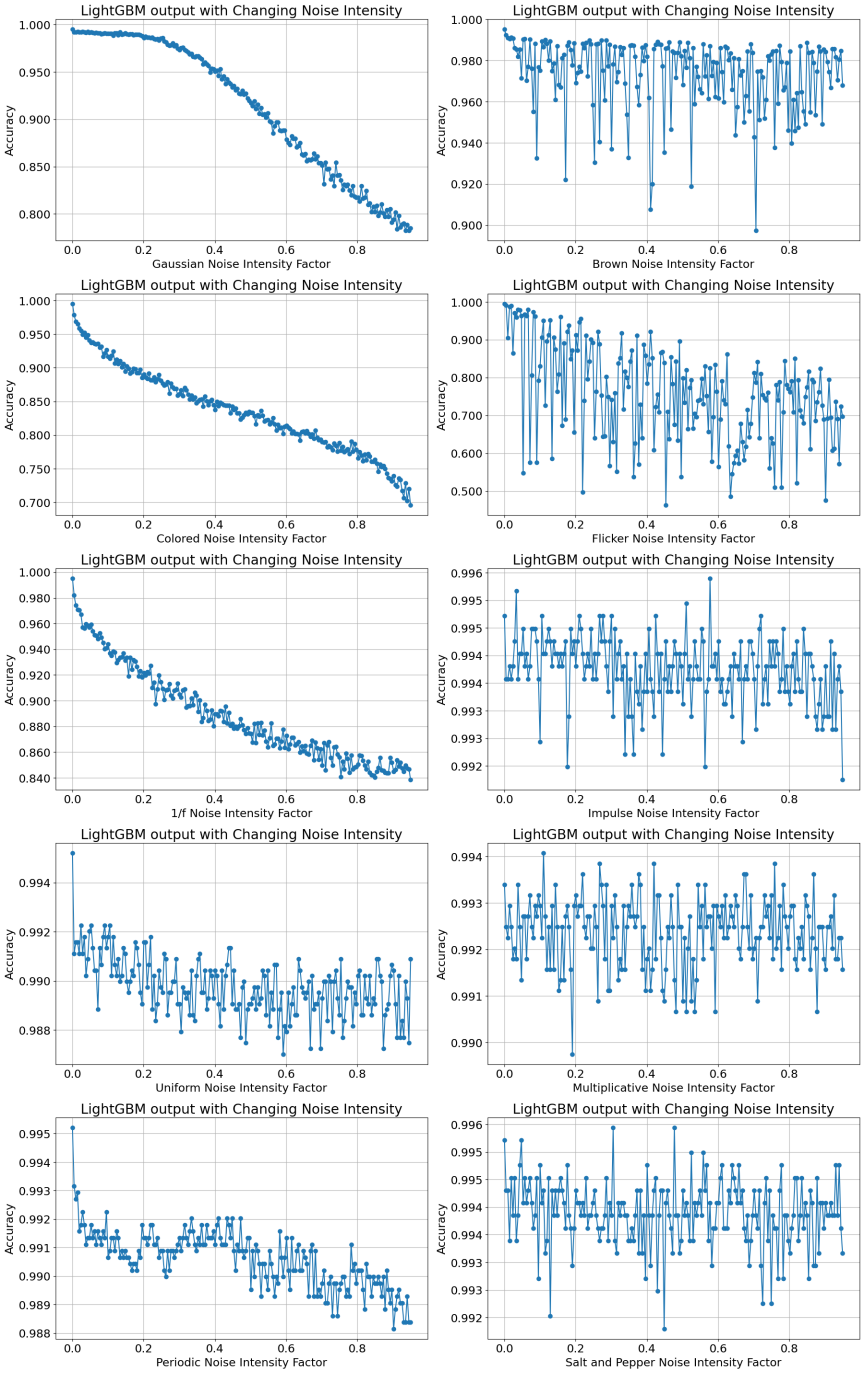
Accuracy of LightGBM with noise intensity values.

The subsequent experiment controlled the intensity of each noise type on a scale from 0 to 95%. It was interesting to determine the threshold limit if it existed for each noise type, which would be where the accuracy of predictions using LightGBM did not downgrade to a large extent. The noise types were added with varying intensities, the accuracy of LightGBM was assessed, and the results are shown in Figure 9. Accuracy and FPR were used as metrics to evaluate the sensitivity of LightGBM to changing noise intensities, since they were straightforward to comprehend and use. Accuracy measured the proportion of precise estimations in the overall predictions, and FPR highlighted Type I error and focused on the frequency of inaccurate classifications of the changeover class. The so-called “easing functions” (https://easings.net/ accessed on 4 January 2024)), as well as self-speaking catchwords from colloquial language, were used to describe the following curve plots.

LightGBM with Gaussian-induced noise significantly dropped its accuracy levels after the noise intensity increased beyond 20%. The changing intensities for Gaussian noise had a “water-pipe-flow” effect and depicted a “concave-down-decreasing” influence on the accuracy values. The intensity factor of 20% can be regarded as the threshold limit for Gaussian noise. The accuracy remained at a high level of about 98% until this threshold limit, after which the accuracy decreased linearly as the intensity factor increased.

However, a visible threshold limit at which the accuracy values were stable was missing for all noise types other than Gaussian noise. The Colored and 1/f noise showed a similar pattern of decreasing accuracy with increasing levels of noise intensity, with the colored noise showing faster convergence and achieving an accuracy of 77.5% at an intensity factor of 0.8, while the 1/f noise showed an accuracy of 85% at an intensity factor of 0.8. The Colored noise had a “long-hair-down” effect and portrayed a decreasing “ease-InOut-Sine” impact on the accuracy values. The 1/f noise had a constant “down-slope” effect and revealed a decreasing “ease-In-Circ” impact on the accuracy.

Uniform noise appeared to have a similar behavior to that of 1/f noise with a “down-slope” effect and a decreasing “ease-In-Circ”. However, while the 1/f noise achieved an 85% accuracy level at a 0.8 intensity factor, the uniform noise exhibited a notably higher accuracy of 98.9% at the same intensity.

Periodic noise followed a pattern resembling the aforementioned curves, showcasing a diminishing “bumpy road” fluctuation in accuracy values beyond 20% of noise intensity. The increasing intensity of Brown noise, however, illustrated a “sky-jump” effect at multiple points of iteration. A constant value in the accuracy levels was observed with deviations of up to 10% of the accuracy for certain data points. Flicker noise showed a linear decline in the accuracy accompanied by large deviations in neighboring data points with a higher intensity of noise. The increase in noise intensity did not cause a continuous decrease in the accuracy levels in the case of Brown, Multiplicative, and Salt-and-Pepper noise. Impulse, Multiplicative, and Salt-and-Pepper noise had a visible “digitization” effect on the accuracy values. In these cases, the range of the accuracy values was quite small, and the data points appeared on discrete levels of accuracy.

The LightGBM-based soft sensor was able to achieve an accuracy in the range of 98% and even 99% for Uniform, Periodic, Brown, Impulse, Multiplicative, and Salt-and-Pepper noise, indicating that these noise families generally had very little impact on the prediction of changeovers, except for certain outlier values in this study. A greater slope of the linear decrease in accuracy values was observed mainly for Periodic and Flicker noise, and in comparison, the linear decrease was marginally smaller for Uniform and Impulse noise. In conclusion, the accuracy was mostly impacted by the intensity of Gaussian, Colored, 1/f, and Flicker noise, as shown in Figure 9.

**Figure 10:**
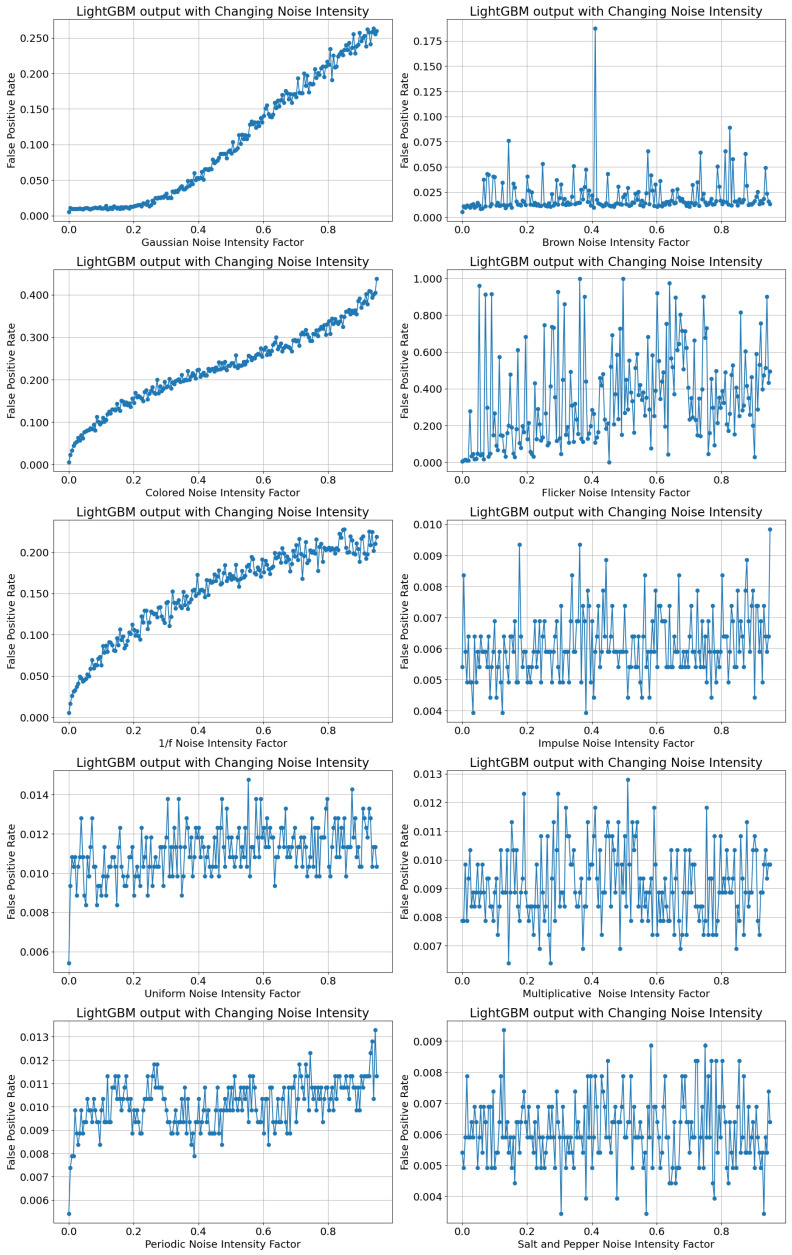
False positive rate of LightGBM with noise intensity values.

Figure 10 shows a similar pattern of outputs and indicates the relation of the FPR of prediction of changeovers using LightGBM with the increase in the intensity of noise. The FPR and accuracy metrics appeared to be mirrored on an imaginary horizontal line in Figure 9 and Figure 10 for Gaussian, Colored, and 1/f noise. This indicated that the LightGBM model required further refinement in the model to classify data induced with Gaussian, Colored, and 1/f noise.

The FPR played a critical role in the analysis of LightGBM’s predictions for detecting negative instances of changeovers or production phases, thereby reducing the frequency of incorrect alerts during changeovers. In situations where the reliability of predictions is significant, a low FPR shows that the model successfully controls the rate of false positives, making it a valuable metric for practical scenarios. These analyses can provide an appropriate balance between the precise classification of changeovers and a reduction in the chances of generating false positives in predictions for each noise type.

## 6. Discussion

There are findings for practitioners that can be directly derived from the interpretation of the figures in the preceding section, which will be discussed in Section 6.1. Additionally, strategic findings will be presented afterward in Section 6.2.

### 6.1. Practical Findings

*Finding 1*: In this case study, it was observed that training with Flicker noise can improve the quality of an ML model. In scenarios where it is likely that a sensor is affected by noise, the Flicker noise tends to make the model more resilient to noisy data and to improve its robustness to environmental influences while predicting changeovers. The false positives and false negatives were further minimized in the output metrics after the addition of Flicker noise to the data. The model generalized well and became less sensitive to random discrepancies due to actual noise being present in the data. The model could comprehend and account for the noise to which it was exposed and, thereby, distinguished between the actual data from the sensor and the noise during the prediction process.

*Finding 2*: Certain types of noises affect the quality of ML models in a negative way more than others. Sensors show the effects of these noises on their data according to the environmental conditions in which they are used. The authors propose that practitioners analyze the environment of their ML tasks for sources of noise before data acquisition. They also advise that practitioners analyze each sensor that they intend to use and that they evaluate the risk of being prone to noise. The sensor selection should then be adapted to these analyses. The concept of measurement uncertainty also considers the effects of stochastic influences, such as noise. Therefore, the selection of sensors should also include an uncertainty consideration concerning the specific environment in which the sensor is used, e.g., according to GUM (see Section 2.1).

*Finding 3*: When it is not possible to adjust the selection of sensors, the impact of noise needs to be mitigated. It was found, for example, that the biggest impact on the metrics of the model was due to Gaussian noise. According to Table 2, this noise was caused by electrical interference and thermal variations. The following mitigation strategies can be derived: (a) According to Table 2, the data can be filtered with Gaussian smoothing, Kalman filtering, or adaptive filtering; (b) the results for Gaussian noise in Section 5 showed that noise intensity below a threshold of 20% can be tolerated. The range of noise intensity in which the specific sensor is affected must be evaluated by the practitioner. Above 20%, the mitigation strategies from (a) apply.

### 6.2. Strategic Findings

*Finding 4*: The ML soft sensor could be calibrated by training with various types of sensor data collected in various case studies to obtain a substantial and comprehensive dataset. The results could be cross-verified using the latest boosting algorithms along with benchmark ML models, such as (SVM) or Random Forest. Cross-validation and bias correction strategies could be used to evaluate the adaptability and efficiency of the model. Choosing the right statistical evaluation metrics and tuning the hyperparameters are additional steps in calibrating soft sensors.

*Finding 5*: A framework for using LightGBM as a soft sensor for detecting changeovers can be designed according to two key possibilities (see Section 3). In Possibility 1, the underlying data from the NC and external sensors are directly given as input into the LightGBM model without induced noise. In contrast, the work discussed and recommended in this article focused on Possibility 2. Here, the data were induced with different types of noise and then passed on to the LightGBM soft sensor, as discussed in Section 3.

Possibility 1 means that when the created model is not good enough, ML techniques such as regularization, hyperparameter tuning, outlier filtering, and feature transformations are applied, or completely new models are created. The sensor data passed to the pipeline have an element of uncertainty, which can go undetected in an ML-based soft sensor. In this case, training the ML model follows a more general black box approach. Although the LightGBM model can predict changeovers in this framework, it fails to quantify the uncertainty of the resulting predictions. A practical scenario in this use case assumes that the LightGBM model predicts changeovers while considering the fact that the data from sensors are of the highest quality and free from noise. Misclassified changeover data can then be adjusted and improved by trying other black-box-based ML methods.

The ML training pipeline follows the general preprocessing and data-filtering techniques. In specific scenarios, a random spike in the data can appear as an outlier value. A data-filtering technique, such as outlier detection and removal, can act as a remedial measure for reducing the uncertainty parameter. The hyperparameters of the LightGBM model can be further tuned to improve the results as a further optimization step. More emphasis on sensor calibration and improving the measurement process is to be considered as a significant step in reducing uncertainty.

Possibility 2 means simulating noise during the creation of an ML model as a “stress test”. It is crucial to note that the inherent data captured in a sensor contain no direct conclusive evidence of the possible type of noise present in them. The process of inducing noise in the sensor data in the LightGBM framework provided further intuition for the quantification of the uncertainty in the predictions from the model. The results from Section 5 show that certain noises, such as Flicker and Brown noise, can be ignored, whereas Gaussian and Colored noise need to be handled further with mitigation techniques, as discussed in Table 2.

It is recommended to further fine-tune the LightGBM model specifically for Gaussian- and Colored-noise-induced data to reduce the incorrect changeover classifications. Grid-based hyperparameter tuning is computationally challenging, and the results of further refinement of the LightGBM parameters are shown in Table A1. Since Gaussian noise was found to be the worst noise for this use case, it can be further inferred that uncertainty in sensor data majorly evolves from electrical interference, temperature variations, and transmission errors in general, as shown in Table 2. Colored noise is observed in motion and location sensors and can be due to mechanical vibrations. The correlated noise patterns also indicate the presence of Colored noise in the observed data. Colored noise can be mitigated using moving average smoothing and spectral analysis, among other methods.

## 7. Summary and Outlook

Overall, this work aimed to assist practitioners in understanding the impacts of noise on ML models. Therefore, the use case of detecting changeover events in manufacturing machines was modeled using an ML soft sensor, LightGBM, and data from an NC and an external indoor positioning system set up for a CNC HERMLE C600 U milling machine. LightGBM was selected as the ML-based soft sensor for the investigation because of its quick training time, minimal memory usage, and excellent prediction efficacy. The key cause of ML soft sensor uncertainty was identified as the noise present in the sensor data, as discussed in Section 2.3.

Although LightGBM is adaptable to a certain amount of noise in data, incorrect data and outliers can result in less-than-ideal model accuracy and FPR. The investigation concluded that the key statistical metrics, namely, Cohen’s Kappa, specificity, recall, average precision, balanced accuracy, FPR, log loss, and others, were affected by the type and intensity of noise. The statistical metrics used for an evaluation are prone to misrepresentation due to noise, thus decreasing the reliability of the model’s performance.

Gaussian, Colored, and 1/f noise distorted the sensor data quality to a greater extent than other noise types did. It is interesting to note that the LightGBM soft sensor could adapt and accommodate moderate noise intensity levels, i.e., up to 20% of Gaussian noise, without having major disruptions in the accuracy and FPR value of the model. Colored and 1/f noise, on the other hand, caused a quick and rapid decrease in the accuracy levels of LightGBM with the constant increase in the respective noise intensities. It is significant to note that for Salt-and-Pepper, Multiplicative, Impulse, Brown, and, to a certain extent, Uniform noise, the trend in accuracy values did not continue to decrease, although there were fluctuations in accuracy at regular intervals in both directions. This pattern of accuracy values was evident even after the continuous increase in the intensity value of the noise after a certain threshold limit. To conclude, the LightGBM ML soft sensor could categorize the specific noise types as having a safe or detrimental effect on the accuracy of predicting changeovers. It also identified a safe threshold limit for each noise type if it existed for the specific noise of interest.

The findings for the initial research questions that were posed in Section 1 can be summarized as follows: Sensor-induced noise types, especially Gaussian and Colored noise, can cause inconsistencies in the data and have a direct impact on the accuracy and reliability of changeover detection. The degree of effectiveness of ML models in detecting changeovers with sensor-induced noise data depends mainly on the type and intensity of noise in the data. Sensor-induced noise can be handled by optimizing the parameters of the ML model through hyperparameter tuning and can be reduced through specific noise mitigation strategies. It was possible to identify a safe threshold of 20% for noise intensity for noise types such as Gaussian noise. A threshold could be identified for noise types such as Colored, Uniform, Periodic, and 1/f noise based on the minimum range of accuracy required for the specific case study. For example, 1/f noise had an intensity threshold of 10% for the ML accuracy to be within the range of 93% in this study. A threshold limit was not critical for certain types of noise, such as Salt-and-Pepper, Multiplicative, and Impulse noise, as the accuracy range was within 99% even with up to 95% scaling of the noise intensity.

Future work will focus on noise mitigation strategies specifically for Gaussian and Colored noise and their effects and influence on the ML soft sensor. Additionally, a preliminary phase of a comparative study was carried out with the DART, XGBoost, HistGradientBoosting, and Random Forest ML algorithms as soft sensors and their scope in reducing and quantifying uncertainty in sensor data processing. For this purpose, following the approach of this study, Monte Carlo simulations with different noise types and noise intensities will be performed. It will be investigated whether these ML algorithms behave similarly to the LightGBM algorithm. It will be taken into consideration for the experimental setup of subsequent work that varying degrees of noise intensity should be applied to the specific numerical input variables based on the underlying use cases that are chosen.

## Figures and Tables

**Table 1:** Feature variables from the NC and indoor positioning sensors.

No.	Variable Name	Description
1	ProgramStatus	Status Code:
		idle: 0
		started: 1
		running: 2
		stopped: 3
		finished: 4
		completed: 5
		interrupted, error, canceled: 6
2	ToolNumber	Tool number
3	PocketTable	Place table for tool changer, number
4	DriveStatus	Drive turned on/off
5	DoorStatusTooling	Tooling door open/closed
6	OverrideFeed	Feed override (0 to 100%)
7	FeedRate	Feed rate (−32,710 to 32,767 m/s)
8	SpindleSpeed	Spindle speed (0 to 10,046 rpm)
9	IndoorGPSx	Indoor positioning system *x*-axis
10	IndoorGPSy	Indoor positioning system *y*-axis

**Table 2:** Comparison of common noise types in sensors.

Noise Type	Source and Observations	Characterization	Mitigation
Gaussian	External electrical interference	Mean	Gaussian smoothing [25]
	Thermal noise from temperature variations	Standard deviation	Kalman filtering [26]
	Transmission errors	PDF follows bell curve	Adaptive filtering: RLS, LMS [27]
Uniform	Environmental conditions	Min and max bounds	Data clipping [28]
	Uniformly distributed quantization noise	Variance	Data smoothing [29]
	Uniformly distributed Jitter noise	PDF constant within a range	Sensor calibration [30]
Salt and Pepper	Random spikes and drops in data	Outliers	Median filtering [31]
	Impulse noise from faulty sensors	Probability of impulse	Linear or cubic interpolation [32]
	Sudden, Intermittent disturbances	Impulse density function	Cleaning outlier [33]
Flicker	Low-frequency noise fluctuations	Flicker noise coefficient	LightGBM modeling [34]
	Thermal fluctuations	Correlation time	Wavelet de-noising or Wiener filtering [35]
	Long-term temporal correlations in noise	Power spectral density	Kalman Filtering [36]
Impulse	Sudden spikes in data	Probability of impulse	Outlier removal [37] Adaptive filtering [38]
	Abrupt noise from electrical sources	Amplitude of impulse	Median filtering [39]
	Random noise spikes due to external disturbances	Impulse distribution	Cubic spline or polynomial interpolation [40]
Multiplicative	Nonlinear effects	Scaling factor	Min-max scaling or z-score normalization [41]
	Affecting the amplitude of the signal	Variability	Logarithmic or power transformations [42]
	Environmental conditions	Statistical properties	Moving average or local smoothing [43]
Colored	Correlated noise patterns	Autocorrelation function	Autoregressive modeling [44]
	Mechanical vibrations	Power spectral density	Moving average smoothing [45]
	Sensor measuring position, velocity, or force	Spectral shape	Spectral analysis: periodo-gram or spectrogram [46] DeepANC [47]
1/f	Flicker noise exhibiting a 1/f power spectrum	Spectral density	Fourier transformation [48]
	Electrical fluctuations	Time variability	Wavelet transformation [49]
	Thermal variations	Long-term correlations	Spectral analysis [50]
Brown	Brownian noise from random walk	Random walk pattern	Random walk modeling [51]
	Random walk–Brownian motion pattern	Autocorrelation function	Savitzky–Golay filtering [52]
	Brownian noise with long-term correlations	Scaling amplitude and time	Data interpolation [53]
Periodic	Periodic noise patterns	Periodic oscillation	Harmonic analysis [54]
	Noise affected by harmonic vibrations	Frequency components	Bandpass filtering [55]
	Periodic disturbances from external sources	Fourier analysis or spectral decomposition	Fourier transformation [56]

## Data Availability

The research data and source code is available on Git Hub: https://github.com/vinaigb/Noise_Uncertainty (accessed on 4 January 2024).

## Abbreviations

The following abbreviations are used in this manuscript:

**AUC-PR**	*Area under the precision–recall curve*
**CNC**	*Computer numerical control*
**DeepANC**	*Deep active noise control*
**FPR**	*False positive rate*
**GAN**	*Generative adversarial network*
**GUM**	*Guide to the Expression of Uncertainty in Measurement*
**KDE**	*Kernel density estimation*
**ML**	*Machine learning*
**NC**	*Numerical control*
**OBerA**	*Optimization of Processes and Machine Tools through Provision, Analysis and Target/Actual Comparison of Production Data*
**SVM**	*Support vector machine*

## Appendix A

In this section, a description of the different noise types is given.

*Gaussian noise*: Gaussian noise represents the inherent stochasticity in data relevant to sensor measurements and serves as a crucial component for modeling and assessing uncertainty. It is frequently used to simulate measurement errors in signal processing and is often referred to as white noise, which denotes that its power spectral density is constant across all frequencies [72]. The random noise is predominantly influenced by the standard deviation σ and the mean μ. Due to its statistical characterization, Gaussian noise is a great option for modeling errors generated in data and analyzing complex systems. The observed phenomenon of data embedded with noise is caused by a number of factors, such as electrical interference and temperature variations [73].

*1/f Noise*: Specific noise signals that have a power spectral density that is inversely proportional to frequency are referred to as 1/f noise. The spectral slope has a value of −1, and the power in each range of frequencies increases as the frequency rises by a factor of 2. The 1/f noise is frequently generated in electronic components and circuits [74]. The spectral density of 1/f noise follows an inverse power law. In addition, 1/f noise is characterized by long-term correlations, which means that the values at different time points are not entirely independent and exhibit temporal variations or non-stationary behavior [75].

*Colored Noise*: Colored noise exhibits distinctive spectral characteristics that may include Gaussian components, power-law behavior, or exponential decay, depending on the specific type of noise. The spectral slope of Colored noise describes how the power or amplitude of the noise signal varies with frequency and depends on the noise signal and distribution features of noise [76]. It has a specific non-zero auto-correlation property and is evident in communication systems and image-processing applications [77].

*Periodic Noise*: The oscillations and repeating patterns in periodic noise occur over an interval of time. It is distinguished by the frequency constituents that make up the harmonics and periodicity of the data [78]. Fourier analysis or spectrum decomposition can be used to quantify the behavior of periodic noise [79].

*Uniform Noise*: The maximum and minimum values of a uniform noise describe the upper and lower limits of a uniform distribution [80]. The probability density function within a given range is a constant and has a null value outside the range. The mean squared error is useful for sensitivity analysis and is crucial in comprehending the symmetric characteristics of uniform noise. It measures the distribution of errors caused by various noise intensities [81].

*Salt-and-Pepper Noise*: Random spikes and drops in data are designated as “salt and pepper”, each with a certain likelihood of occurrence. The maximum and minimum values are utilized as the particular positive and negative spikes in the data [82]. The probability of salt and pepper in data is described by an impulse density function [83].

*Flicker Noise*: The Flicker noise coefficient is a parameter that describes how strong the Flicker noise is in data. The correlation time quantifies the period of time during which the noise frequencies continue to correlate and is characterized by long-term correlations [84]. Flicker noise is denoted by a pink noise power-law distribution for its power spectral density [85].

*Impulse Noise*: The frequency and magnitude of spikes depend on the likelihood of Impulse noise, which appears arbitrarily in data. The degree of abrupt disruption or interference in the signal is dependent on the magnitude of the Impulse noise [86]. Impulse noise can follow different distributions, such as uniform or Gaussian distributions, based on the application [87]. In this article, the term “Impulse noise” was used synonymously with impulsive noise.

*Multiplicative Noise*: The amplitude of actual data is affected by the scaling factor introduced by Multiplicative noise. The degree to which noise modifies the actual data depends on the scaling factor’s variability [88]. Multiplicative noise is characterized by its impact on the statistical properties of the data—especially the variance [89].

*Brown Noise*: Brown noise has a random walk pattern and is generated by accumulating randomized steps of errors within the data. It follows an auto-correlation function, which indicates that at various time intervals, the noise intensities are correlated [90]. The intensity and behavior of brown noise on various time scales are influenced by its scaling characteristics [91].

## Appendix B

The noise type and the controlled parameter details are given in the following.

The Gaussian, Flicker, Colored, 1/f, and Brown noise had a mean value of zero and a standard deviation of one as parameter values. The Colored noise had 0.8 as its power value to control the strength of the noise and a sampling rate of 1 to control the data sampling. The power and sampling rate parameters were then used with a low-pass filter to generate Colored noise. The 1/f noise was generated using a Butterworth filter with the critical frequency of the filter set to 1/50. The Uniform noise had a lower bound of −1 and an upper bound of 1 as parameter values. The Salt-and-Pepper noise had a random sampling of [−1, 0, 1] values with corresponding parameter probability values of *p* = [0.05, 0.9, 0.05]. The Impulse noise had a random sampling of [−1, 1] values with corresponding parameter probability values of *p* = [0.9, 0.1]. The Multiplicative noise had a lower bound of 0.1 and an upper bound of 0.9 as the parameter values. The Periodic noise had a frequency parameter of 10 for generating the sine wave.

## Appendix C

The values after hyperparameter tuning for LightGBM are mentioned in Table A1.

**Table A1:** Hyperparameter tuning for LightGBM.

Hyperparameter	Values
Learning Rate	0.15
Max Depth	5
Num Leaves	30
Min Child Samples	20
Subsample	0.7
Colsample bytree	0.8
Reg Alpha	0.0
Reg Lambda	0.03
Bagging Fraction	0.8
Boosting Type	dart
Min Split Gain	0.05
N Estimators	100

## Appendix D

The F1 score values obtained using LightGBM for different noise types are detailed in Table A2.

**Table A2:** F1 scores of LightGBM with multiple types of noise.

Noise Type	F1 Score
Flicker	0.99952
Brown	0.99934
Gaussian	0.98739
Salt and Pepper	0.99428
Multiplicative	0.99370
Colored	0.98860
Periodic	0.99360
1/f	0.99156
Uniform	0.98983
Impulse	0.99479
